# Time Course of High-Energy Phosphate Depletion During Cold Storage of Human Heart Grafts Using the Celsior Solution

**DOI:** 10.3389/ti.2024.12994

**Published:** 2024-07-12

**Authors:** Frank Kober, Thierry Caus, Alberto Riberi, Yann Le Fur, Monique Bernard

**Affiliations:** ^1^ Aix-Marseille Univ., CNRS UMR 7339, Centre de Résonance Magnétique Biologique et Médicale (CRMBM), Marseille, France; ^2^ Department of Cardiovascular Surgery, La Timone University Hospital Center, Marseille, France; ^3^ Department of Cardiac Surgery, Amiens Picardie University Hospital Center, Amiens, France

**Keywords:** magnetic resonance spectroscopy, phosphorus, heart transplant, high-energy metabolism, cold storage

## Abstract

The aim of this study was to provide insight into high-energy phosphate compound concentration dynamics under realistic clinical cold-storage conditions using the Celsior solution in seven heart grafts discarded from transplantation. The hearts of seven local donors (three males, four females, age 37 ± 17 years, height 175 ± 5 cm, weight 75 ± 9 kg) initially considered for transplantation and eventually discarded were submitted to a Magnetic Resonance Spectroscopy observation in a clinical Magnetic Resonance Imaging scanner over at least 9 h. The grafts remained in their sterile container at 4°C during the entire examination. Hence, Phosphocreatine (PCr), adenosine triphosphate (ATP), inorganic phosphate (Pi) and intracellular pH were recorded non-destructively at a 30-minute interval. With the ischemic time Ti, the concentration ratios decreased at PCr/ATP = 1.68−0.0028·Tis, Pi/ATP = 1.38 + 0.0029·Tis, and intracellular pH at 7.43–0.0012·Tis. ATP concentration remained stable for at least 9 h and did not decrease as long as phosphocreatine was detectable. Acidosis remained moderate. In addition to the standard parameters assessed at the time of retrieval, Magnetic Resonance Spectroscopy can provide an assesment of the metabolic status of heart grafts before transplantation. These results show how HEPC metabolites deplete during cold storage. Although many parameters determine graft quality during cold storage, the dynamics of HEPC and intracellular pH may be helpful in the development of strategies aiming at extending the ischemic time.

## Graphical Abstract

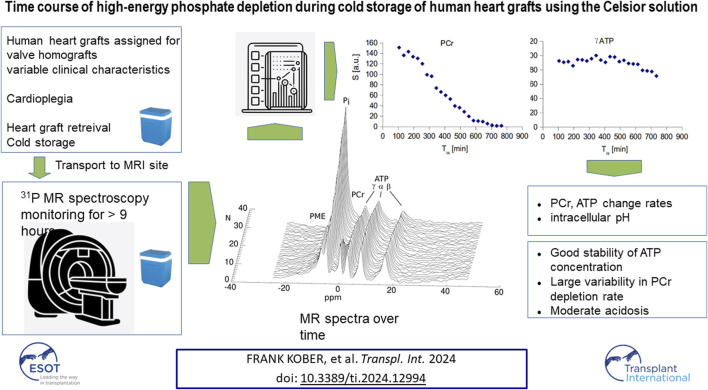

## Introduction

Evaluation of heart grafts using non-invasive techniques before transplantation into the recipient appears as a useful addition to the current standard clinical practice, in which no objective examination of the graft is performed immediately before transplantation. A large variety of destructive and non-destructive biomarkers have been proposed in both clinical and preclinical settings [1]. Despite emerging machine-perfusion beating-heart storage devices [2–4], cardioplegic arrest followed by cold storage of the graft currently remains the most widely used method. In both warm and cold storage situations, an objective evaluation before transplantation could contribute to increasing transplant safety on the one hand and to widening the donor pool on the other. One important indicator of graft quality is given by high-energy phosphate compound (HEPC) concentrations [5, 6] that decrease in the absence of nutriment and oxygen supply via perfusion, and it is one purpose of preservation solutions to prevent their rapid depletion. These metabolite concentrations can be assessed in a completely non-destructive way using phosphorus-31 Magnetic Resonance Spectroscopy (^31^P MRS). Using MRS, the quality of graft preservation with different cardioplegic solutions has been assessed in animal hearts [3, 7–10], and metabolite preservation before graft implantation has been analyzed in human hearts [5, 6] during cold storage and, more recently, during warm storage under machine perfusion [4]. However, little is known about the influence of storage time on the metabolic preservation quality of human heart grafts in a clinical context, where an estimation of metabolic depletion rates in human grafts could help strengthen safety. The purpose of this work was to assess the time course of HEPC alterations during cold storage of human heart grafts. This was possible using hearts that were discarded from transplantation based on clinical donor criteria but that underwent the same retrieval and cold storage procedure as transplanted grafts for the purpose of this study.

## Materials and Methods

No rupture of sterility or low temperature was caused to the grafts by this non-destructive assessment. The study was approved by the University Hospital’s ethics committee “Comité consultatif de protection des personnes dans la recherche biomédicale—Marseille 2” (Authorization #99/16) and conducted in accordance with the ethical standards laid down in the 1964 Declaration of Helsinki and its later amendments. The donors’ next of kin gave their written informed consent to the heart graft retrieval. This retrospective dynamic data analysis was carried out in 2024 on Magnetic Resonance data acquired from 1999 to 2001. According to current rules confirmed by the French Agence de Biomédecine, this analysis is therefore exempted from additional specific ethical approvals.

### Donors

The hearts of seven local donors (three males, four females, age 37 ± 17 years, height 175 ± 5 cm, weight 75 ± 9 kg) initially considered for transplantation were clinically evaluated before retrieval. Based on echocardiography data (Left-Ventricular Ejection Fraction < 50%) and/or the level of administered inotropes (Adrenalin > 2 mg/h) administered to the donor, all grafts used in this study were eventually qualified unsuitable for heart transplantation, but they were retrieved for valve graft excision.

### Retrieval and Transport

All hearts were arrested and preserved with Celsior^®^ [11] cardioplegic solution at 4°C. After excision, the grafts were placed in sterile plastic bags filled with Celsior^®^ solution. The bags were sealed and inserted in plastic jars containing physiologic NaCl solution. The jars were put on ice in an insulated container and transported to the MRI/MRS facility. The average ischemic time at arrival on the MR site was 124 ± 71 min.

### MR Protocol

The ice container was positioned on a commercially available ^31^P/^1^H surface radiofrequency coil in the magnet of a Siemens 1.5 T clinical MR system. This way, sterility and stable temperature conditions could be ensured at any time of the examination. Automated localized map shimming was performed. A stack of multi-slice T1-weighted proton MR images was acquired to determine the position of the graft within the container. A single-pulse free induction decay spectroscopy sequence (TR = 10 s, 32 averages, duration 5 min) was used to obtain global full-relaxed ^31^P-MR spectra at 25.9 MHz. The acquisition was repeated every 30 min as long as the scanner was available. Since the majority of grafts arrived at week nights, the time was limited to 9 h, except for one examination that could be left running for 13 h. The time between cardioplegic arrest and the beginning of the MR examination was recorded.

### Data Processing

All spectra were quantified without user interaction by the AMARES time-domain fitting routine part of the MRUI software package [12]. The signal ratios of phosphocreatine (PCr)/adenosine triphosphate (ATP), PCr/P_i_, P_i_/ATP and phosphomonoesters (PME)/ATP were calculated. The γATP resonance was used to represent ATP. The intracellular pH value (pH_i_) was obtained by Kost’s formula [13] using the chemical shift difference between the PCr and the P_i_ resonances. Linear regression was used as a simple approach to characterize dynamic alterations of metabolite signals (PCr, P_i_) and pH_i_ over time. The slopes obtained with linear regression were expressed as percent decrease with respect to the extrapolated signal value at cardiac arrest. No absolute quantification of metabolite levels could be performed, since the position, size and shape of the grafts varied across the exams.

## Results

**FIGURE 1:**
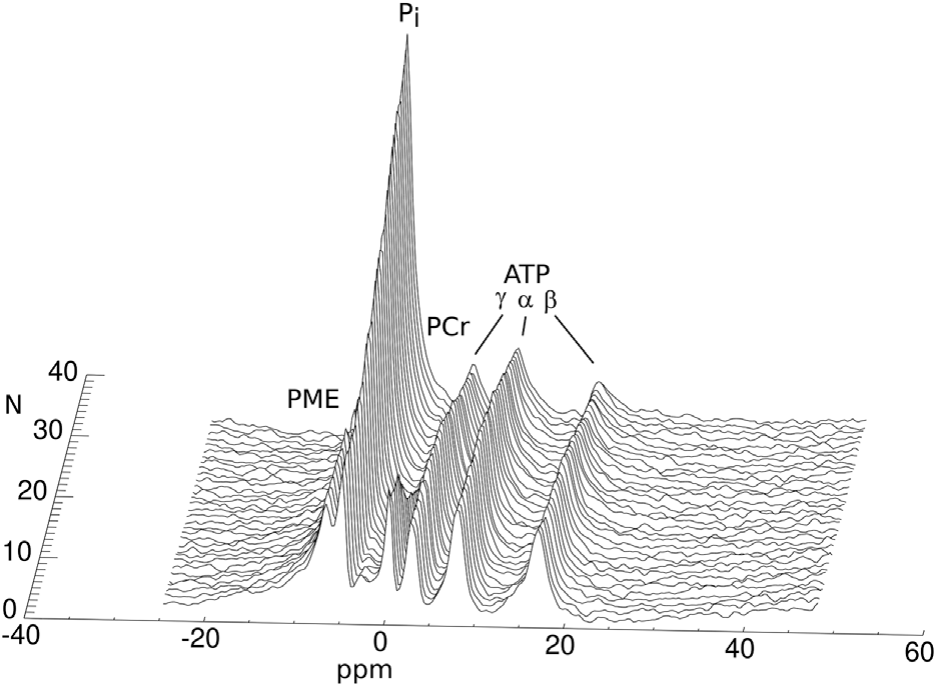
Phosphorus MRS spectra of a human heart graft discarded from transplantation during cold storage. The spectra were acquired with a 30-minute interval between them (N: number of the acquisition). The annotated resonances in each spectrum represent the concentrations of the different HEPC.

**FIGURE 2:**
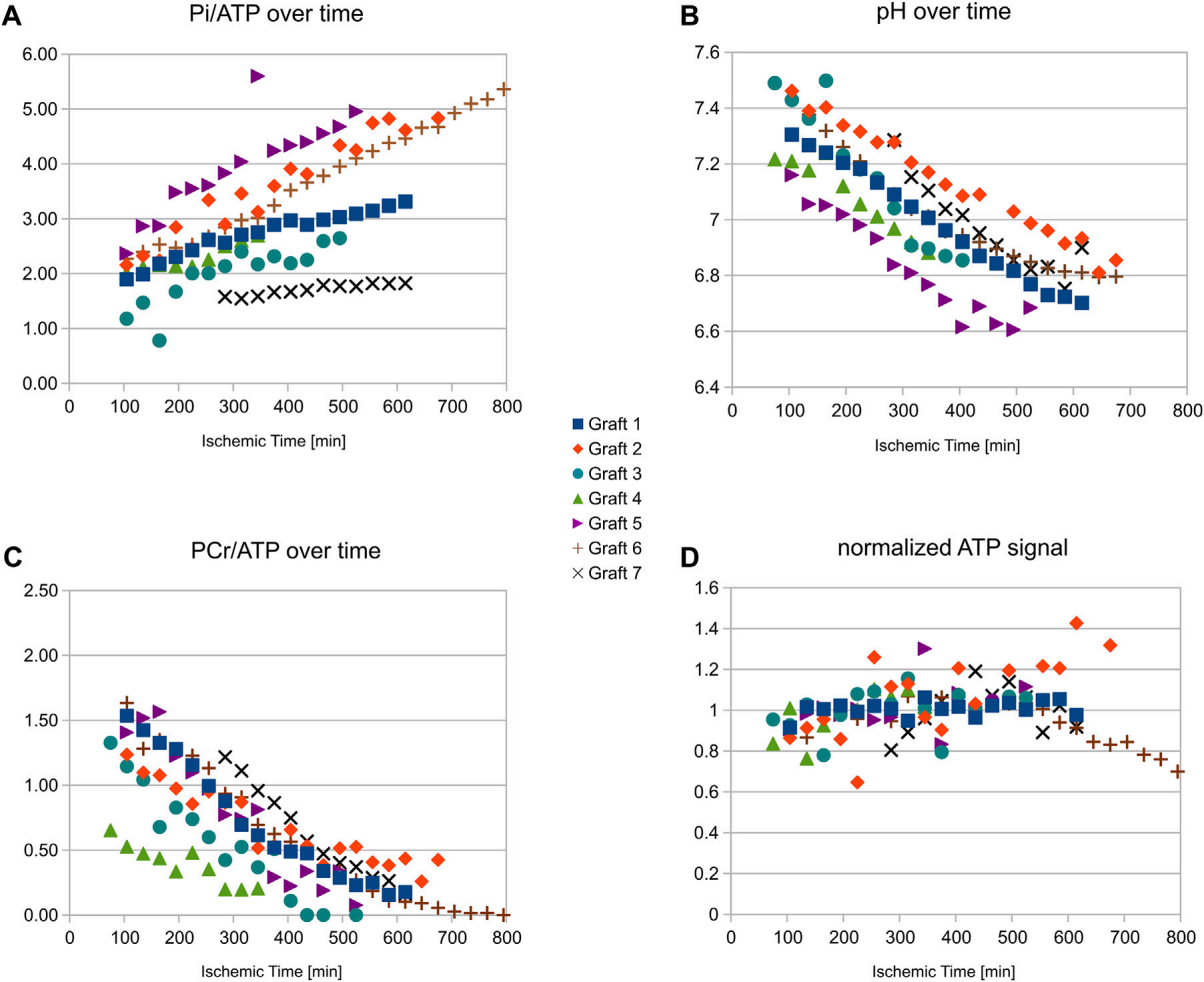
Resonance amplitudes for different HEPC and intracellular pH versus ischemic time during cold storage for all grafts studied. Note that the grafts arrived with different transport times on site. Despite a relatively large variability in the concentration ratios (especially for P_i_ **(A)**), the measurements show good stability of the ATP **(D)** concentration as long as PCr **(C)** was not fully depleted (always more than 9 h in this study) as well as moderate acidosis **(B)**. The stronger concentration variability in some grafts was caused by variable distances of the grafts in their container from the MR radiofrequency antenna.

An example stack plot of spectra from the graft observed during 13 h as a function of time is displayed in Figure 1. Figure 2 shows the signal amplitudes of PCr, P_i_, γATP and phosphomonoesters (PME) as a function of time after cardioplegic arrest for all examined grafts. The graphs account for the variations in arrival time on site. For visualization, three data points from misfitted spectral resonances of graft number 2 were removed. Figure 2 also shows the intracellular pH as long as PCr was measurable.

In the seven grafts, PCr signal, normalized to that at the time of retrieval, decreased by 10.3% ± 1.9%/hour and P_i_ increased by 12.6% ± 5.0%/hour. From the graft followed for 13 h (shown in Figure 1) it can further be seen that ATP levels were stable until PCr was depleted, and PCr depletion did not occur in the other observed grafts, i.e., before 9 h of storage. PCr decrease and P_i_ increase were well represented by the linear regression over 9 h. The concentration ratios PCr/ATP and Pi/ATP can, therefore, be expressed as a linear function of the ischemic time *T*_is_ [min]: PCr/ATP = 1.68-0.0028·*T*_is_, Pi/ATP = 1.38 + 0.0029·*T*_is_. Intracellular pH obtained from the chemical spectral shift of Pi also showed a shallow linear decrease over time: pH = 7.43–0.0012·*T*_is_, indicating good stability of the acidic level.

## Discussion

We have measured the time course of changes in HEPC metabolite concentrations in seven human heart grafts during cold storage. HEPC metabolites are known contributors to the graft quality during transplantation. Depletion of high energy phosphates during ischemia is linked with harmful injury in the ischemic myocardium [6], and levels of high energy phosphate have been correlated to the recovery of function after transplantation both in animal models and human studies [6, 14–16]. In a more recent study by Föll et al. [17], the ischemic storage time was also shown to correlate with regional wall motion after transplantation.

Signal variations show that on a timescale typical for cold storage duration in heart transplantation (maximum 4 h), HEPC metabolites in human heart grafts are well preserved. Although PCr was decreasing rapidly, the ATP levels in all six example heart grafts were stable for at least 9 h following cardioplegic arrest and storage with Celsior. This indicates that in current clinical practice using these conditions of preservation, the level of ATP is well preserved over a long cold storage duration. ATP is indeed related to numerous cell structure mechanisms and to contractility, and its depletion associated with irreversible loss of precursors. These results also show a moderate acidosis of the grafts at pH 6.8 after 9 h of ischemia. Earlier studies carried out in animal hearts during ischemia with cardioplegia have shown that the intracellular pH is dependent on the pH and on the buffering capacity of the preservation solution [10]. While the heart is able to recover from mild acidosis, severe acidosis would induce cell necrosis.

We have used ^31^P Magnetic Resonance Spectroscopy for this analysis. This tool proved to be particularly useful, since the entire exam could be accomplished with preserved sterility and temperature with *in situ* determination of metabolites and intracellular pH. The dynamic measurement allowed us to derive change rates for the major compounds ATP, PCr and P_i_ and for the intracellular pH. These rates can be considered as characteristic for the particular group of grafts assessed here. As a difference with earlier work by our group and others [5, 6], all grafts studied here were discarded from transplantation based on their clinical score. This leaves some uncertainty on the initial metabolic status of the grafts before retrieval, since grafts with lower clinical scores were shown earlier to have lower PCr/P_i_ concentration ratios [5]. This likely explains the relatively low initial PCr/ATP and high Pi/ATP ratios found even when linearly extrapolating them back to the moment of retrieval.

The influence of ischemic time on HEPC metabolism in heart grafts has been evaluated earlier by van Dobbenburgh and coworkers [6] in a study correlating functional performance and metabolic status before transplantation in 25 heart grafts arrested with St Thomas cardioplegic solution. One has to keep in mind that van Dobbenburgh et al. assessed grafts that were actually transplanted at relatively short ischemic times (<2 h), allowing them to acquire only one time point per graft. The authors also reported a correlation of metabolism and the variable ischemic time (*T*_is_ [min]) of each graft, although only one time point per graft was available. Their constructed decreases over time were PCr/ATP = 1.31−0.0039·*T*_is_, Pi/ATP = 0.26 + 0.0064·*T*_is_ and pH = 7.66-0.0040 ·*T*_is_ and can be compared with our regressions: PCr/ATP = 1.68−0.0028·*T*_is_, Pi/ATP = 1.38 + 0.0029·*T*_is_, and pH = 7.43−0.0012·*T*_is_. During our nine-hour observation period, PCr, Pi and pH, therefore, decreased slower than in the <2 h period observed in van Dobbenburgh’s mult-organ study. Initial Pi, as calculated by linear extrapolation to the moment of explantation, was, however, found much higher in our study. These differences may be related to the low clinical scores reported for the grafts used here. In our study, time courses were obtained from each graft individually over time periods of 9 h. The change rates may also vary as a function of the storage solution (here: Celsior), which was shown earlier to have an influence on metabolite levels during reperfusion in rat hearts [14].

Similarly to observations reported by van Dobbenburgh, we observed a relatively large variation in both PCr/ATP and, especially, Pi/ATP ratios of the donor hearts as well their change rates. These differences may be caused by the condition of the donor, the use of pharmacological therapy, and the quality of cardioplegic arrest and hypothermic preservation before arrival on site. The low number of grafts available for this study, however, did not allow us to evaluate statistical correlations with these parameters.

## Limitations

For obvious reasons, the number of grafts at our disposal was limited by the availability, since only a single center contributed to this study. The standard deviation of metabolite change rates was therefore relatively high (in the 20% range). We report these results to give an estimate of the order of magnitude of metabolic changes during cold storage. Further observations will be needed to strengthen these results and to generalize the conclusions with sufficient confidence.

## Conclusion

We have non-destructively analyzed the long-term behavior of high-energy phosphate compounds in human heart grafts. Phosphorus MRS proved to be a useful tool for such a non-destructive analysis. Although many parameters affect graft quality during cold storage, the dynamics of HEPC and intracellular pH may be helpful in the development of strategies aiming at extending the ischemic time. Beyond cold storage, MRS may also provide interesting additional information for evaluating grafts during or after machine-perfused graft storage in comparison.

## Data Availability

The raw data supporting the conclusions of this article will be made available by the authors, without undue reservation.
